# Development of Nationwide Excess Lifetime Cancer Risk Evaluation Methods with Comprehensive Past Asbestos Exposure Reconstruction

**DOI:** 10.3390/ijerph18062819

**Published:** 2021-03-10

**Authors:** Dongmug Kang, Seung Ho Lee, Yoon Ji Kim, Tae Kyoung Kim, Ju Young Kim, Youngki Kim

**Affiliations:** 1 Department of Preventive and Occupational & Environmental Medicine, Medical College, Pusan National University, Yangsan 50612, Korea; kangdm@pusan.ac.kr (D.K.); harrypotter79@pusan.ac.kr (Y.J.K.); 2 Department of Occupational and Environmental Medicine, Pusan National University Yangsan Hospital, Yangsan 50612, Korea; cjsfhleo10@pusan.ac.kr; 3 Environmental Health Center of Asbestos, Pusan National University Yangsan Hospital, Yangsan 50612, Korea; jooykim@pusan.ac.kr; 4 Medical Research Institute, Pusan National University, Yangsan 50612, Korea; 201882126@pusan.ac.kr

**Keywords:** asbestos, past, excess, lifetime, cancer, risk, occupational, environmental, exposure, Korea

## Abstract

Although exposure to asbestos via various routes has been acknowledged, comprehensive exposure and risk assessment methods have not been developed at the national level. We conducted a study to reconstruct comprehensive past asbestos exposure estimations and to suggest a method to calculate the Excess Lifetime Cancer Risk (ELCR) of Koreans. The past occupational exposure reconstruction was conducted by rebuilding the previous general population job-exposure matrix (JEM). The para-occupational and household exposure estimation was based on the pooled analysis of data from other countries as well as Korea. The neighborhood exposure from occupational sources by distance was estimated by the exponential decay model. As a result, 141 JEM exposure groups across four periods including ~79, the 80s, 90s, 2000s with a ratio of 2.0:1.0:0.5:0.05 were reconstructed. The para-occupational and household exposures were 11% and 1% of the JEM respectively. The environmental exposure source concentration from outside occupational exposure was 2.5% of the inside concentration. The ratio of the concentration of environmental exposure source (C_0_) to distance d (C_d_) was $exp^{-kd}\;$ with a decay constant k of 6.834. The mean concentrations (f/cc) were 2.28 × 10^−3^ for outdoor, 4.65 × 10^−5^ for indoor, 1.95 × 10^−2^ for transportation activity, 4.44 × 10^−2^ for agricultural activity, and 4.68 × 10^−2^ for daily life activity in naturally occurring asbestos areas. Indoor and outdoor asbestos concentrations from living in a slate roof house were 1.73 × 10^−6^ and 2.70 × 10^−8^, respectively. For improved generalizability, validity, and applicability of the proposed method, further studies on each route with real assessments and experiments are required.

## 1. Introduction

All forms of asbestos are well known to cause asbestos-related diseases (ARDs) including benign diseases such as asbestosis and pleural plaques (thickening), and malignant cancers such as malignant mesothelioma (MM), lung cancer (LC), laryngeal cancer, and ovarian cancer [1]. The exposure routes to asbestos via various sources including occupational, para-occupational, neighborhood, and household have been acknowledged since the 1960s [2,3,4]. The major sources of occupational asbestos exposure are asbestos mines, asbestos cement and textile factories, and workplaces handling asbestos products including shipyards and construction sites. Indirect occupational (para-occupational) exposure could occur among workers who do not directly deal with asbestos. Asbestos emission from occupational sources could result in neighborhood exposure for people living close to the sources. Naturally occurring asbestos (NOA) and living in an area having an asbestos slate roof are contemporary asbestos exposure situations [5]. As occupational exposure can also cause household exposure to families from the asbestos-contaminated working clothes of exposed workers, occupational exposure assessment may be the starting point to estimate neighborhood and household exposures.

Because ARDs have long latency periods from at least 10 years to as long as 50 years [6], it is necessary to reconstruct previous exposure routes to establish their link with current ARDs. The job-exposure matrix (JEM) is one way to estimate past occupational exposure with limited information on the general working population [7]. Although the reconstruction of the Korean JEM extended to combinations of industrial and occupational groups [8], the missing information in each cell and period before 1979 needs to be filled to function as starting points for other exposure estimations. The neighborhood exposure estimation of the general population at the country level requires information regarding nationwide environmental exposure sources [9]. While the emission from inside to the outside of an exposure source could be checked by a simultaneous assessment of both sides, it is hard to find such a study. When the ambient air concentration of a neighborhood exposure source point is known, dispersion to the surrounding area could be estimated by a mathematical model such as the exponential decay by distance model or modeling using meteorological data [10,11]. Nationwide comprehensive asbestos exposure estimation needs tremendous effort including the collection of data and exposure reconstructions via various exposure routes.

Exposure assessment and estimation are used for epidemiological studies and compensation purposes. While the reference dose leading to benign ARDs has not been suggested so far, exposure estimations for increasing cancer risk could be useful. While the Helsinki criteria indicated a doubling of lung cancer risk, with a cumulative exposure dose of 25 fiber-years/cc [12], recent studies lowered the cumulative exposure dose for lung cancer risk [13]. As the Helsinki criteria mainly suggested a cut-off value, quantitative risk assessment methods such as Excess Lifetime Cancer Risk (ELCR) of asbestos proposed by the US Environmental Protection Agency (EPA) give more information to the public [14]. We conducted a study to reconstruct past asbestos exposure estimations from various exposure routes and to suggest a method for calculating the Korean ELCR.

## 2. Materials and Methods

### 2.1. Korean Asbestos JEM Reconstruction

Two methods were used to fill in the blanks of the recent Korean asbestos JEM containing 141 combinations (exposure groups) of industries and occupations over three decades (the 80s, 90s and 2000s). The first was to apply a decreasing trend from decade to decade using a ratio directly involving the production of asbestos contacting material (ACM) or dealing in ACMs, which was suggested by a previous study [15]. Data reanalysis using the available data without blanks from all three decades was conducted to build ratios between decades, and blanks were filled with the value computed using the ratio between the adjacent decades. Since almost all studies assessing the ambient asbestos concentration were conducted after the 1980s, the data before 1979 was estimated at double that of the 1980s which was a conservative value arrived at by the previous study for past exposure estimation [16]. The second method was to apply the same or constant values for the jobs (exposure groups) assuming the same working conditions through the decades under consideration, which in turn were related to asbestos-contaminated talc, processes using insulating felt or friction material, and working under/beside ACMs such as slate roofs.

### 2.2. Para-Occupational Exposure Estimation

A meta-analysis was performed on literature from Korea and the databases of other countries to determine the concentrations due to para-occupational exposure. A pooled analysis of studies until 2016 was conducted using PubMed and the Research Information Sharing Service (RISS, Daegu, Korea) with the terms of (asbestos) and (para-occupational or concentration or assessment or exposure or indirect exposure or air or lung cancer or malignant mesothelioma or asbestosis or diffuse pleural thickening or colleague at work or co-worker). From the 4260 articles selected using the search terms “asbestos” and (para-occupational or colleague at work or co-worker), 17 studies were included for the pooled analysis (Appendix A). Literature lacking asbestos concentration values (mean and standard deviation) was excluded from the pooled analysis.

### 2.3. Neighborhood Exposure from Occupational Asbestos Exposure Sources Using JEM

Only one study which conducted a simultaneous assessment of the inside and outside (by distance) of a factory was found despite an elaborate literature review [11]. Korea had no valid nationwide meteorological and geographic data before the 1990s. Therefore, we could not use simulation tools that needed specific meteorological data for past neighborhood exposure reconstruction from point environmental sources. Hence, we applied an exponential decay by distance model to formulate the required equations.

### 2.4. Neighborhood Exposure from NOA and Living under the Slate Roof House

Because NOA exposure estimations need specific location and exposure situations including agricultural activities, vehicle use, and other activities, previous studies regarding activity-based sampling (ABS) in NOA areas in Korea were pooled and analyzed. A total of 2194 articles located using the search terms (asbestos mine or naturally occurring asbestos) in RISS and Google Scholar until December 2019 were reviewed and 16 articles were shortlisted for analysis. Also, pooled analysis of living under slate roofs and non-occupational roof renovation activities were conducted. A total of 419 articles were identified using the search term “asbestos slate” in RISS, and Google Scholar until December 2019 and eight were selected after review.

### 2.5. Household Exposure from Occupationally Exposed Family Members

A pooled analysis of 3156 articles with studies until 2016 was conducted using PubMed and RISS with the terms of (asbestos) and (home or house or concentration or assessment or exposure or indirect exposure or secondhand or laundry or the wash or washing or family or familial or wife). The final 11 studies were included in the pooled analysis (Appendix B).

### 2.6. ELCRs

The airborne asbestos inhalation exposure algorithm was based on the 1992 PTI HRA [17]:

EC = (Ca × ET × EF × ED)/AT

where, EC = Chronic Exposure Concentration (averaged over a 70-year lifetime) [f/mL], Ca = Asbestos Concentration in fibers per cubic centimeter (f/cc), ET = Exposure Time in hours/day, EF = Exposure Frequency in days/year, ED = Exposure Duration in years, AT = Average Time of 24 h/day × 365 days/year × 70 years (lifetime).

The ELCRs calculations were made using the equation described in EPA risk assessment guidance document [18].

ELCR = EC × URF

where, ELCR = Excess Lifetime Cancer Risk, URF = Unit Risk Factor for inhalation of asbestos [0.23 (f/mL)^−1^].

## 3. Results

### 3.1. Korean Asbestos JEM Reconstruction

Results from the data reanalysis without blanks in any of the 3 decades of the recent Korean asbestos JEM are shown in Table 1. The mean concentrations (f/cc) 80s, 90s, and 2000s were 1.41, 0.57, and 0.06 with ratios of 1.00, 0.40, and 0.05 respectively.

To make conservative and simple calculations with the ratio of asbestos concentrations between the 80s and ~1979 as 1.0 vs. 2.0, we used the ratios of ~1979:80’:90’:2000~ as 2.0:1.0:0.5:0.05. Filling of the jobs (exposure groups) directly involved in the production of ACMs in the recent Korean asbestos JEM was done using the decreasing ratio by decades. Other jobs (exposure groups) were filled with the same data as adjacent values. The final reconstructed Korean asbestos JEM is shown in Appendix C (reconstructed Korean asbestos JEM).

### 3.2. Para-Occupational Exposure Estimation

Eleven percent of the direct occupational exposure was the para-occupational exposure level as shown by the pooled analysis. When the JEM of the directly exposed job (exposure group) was known, then 11% for indirect exposure in the same workplace during the period could be applied.

### 3.3. Neighborhood Exposure from Occupational Asbestos Exposure Sources by Distance

**Figure 1:**
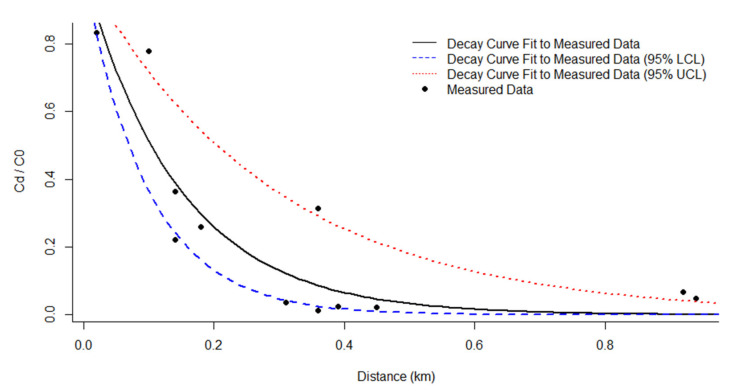
Exponential decay model of ratio $C_{d}/C_{0}$ in ambient air by distance. Where, *d*: the distance (km), *C_d_*_:_ the asbestos concentration at distance *d* (f/cc), *C*_0_: the asbestos concentration at distance 0 (f/cc).

Mean air concentrations inside and outside a factory were 2.4003 f/cc and 0.0601 f/cc (SD 0.03454 f/cc), and outside concentration was 2.5% (SD 1.1%) of the inside reading. The exponential decay by distance model was recalculated using the previous study and was based on the equation below [11]. The constant k was 6.834 with 95% confidence interval of 3.466~10.222 (R^2^ = 0.81) (Figure 1):

$$C_{d}=\;C_{0}\times exp^{-kd}$$

$$C_{d}/C_{0}=\;exp^{-kd}$$

where, *d*: the distance (km), *C_d_*: the asbestos concentration at distance *d* (f/cc), *C*_0_: the asbestos concentration at distance 0 (f/cc), *k*: the overall fiber decay constant = 6.834.

When the inside concentration of a specific place at a specific time was known, the *C*_0_ would be estimated as 2.5% of the inside concentration, and *C_d_* might be estimated by the above exponential decay equation. When the inside concentration was unknown, the revised JEM could be used.

### 3.4. Neighborhood Exposure from NOA and Living under the Slate Roof House

When the specific location and activities of a person in the NOA areas were known, the specific concentrations acquired by this study could be applied. Otherwise, the representative value of pooled analysis could be applied. The mean concentrations (f/cc) were 2.28 × 10^−3^ for outdoor, 4.65 × 10^−5^ for indoor, 1.95 × 10^−2^ for transportation activity, 4.44 × 10^−2^ for agricultural activity, and 4.68 × 10^−2^ for daily life activity in the NOA area. Estimated data according to the hours of staying home and by age could be applied to the person who had lived in the NOA area or under a slate roof house. Indoor and outdoor asbestos concentrations from living in a slate roof house were 1.73 × 10^−6^ and 2.70 × 10^−8^, respectively.

### 3.5. Household Exposure from an Occupationally Exposed Family Member

One percent of the occupational exposure from exposed family members was the household exposure level as per the pooled analysis. When the JEM of the exposed family member and the cohabitant period were known, then the 1% level during the period could be applied.

### 3.6. Cancer Risk Calculation and Risk Grade Determination

The ELCRs calculations were made using below equation.

$$ELCR=\overset{6}{\underset{i=1}{\sum }}[(EPC_{i}\times TWF_{i})\times IUR_{i}]$$

where, *EPC* = Exposure Point Concentration, *TWF* = Time Weight Factor, *IUR* = Inhalation Unit Risk [0.23 (f/mL)^−1^], *i* = 1 (occupational exposure), 2 (para-occupational exposure), 3 (neighborhood exposure from occupational exposure sources by distance) 4 (neighborhood exposure from naturally occurring asbestos, NOA), 5 (neighborhood exposure living under the slate roof house), 6 (household exposure from an occupationally exposed family member).

Final risk might be divided into four groups (with ELCRs) of very high (1.0 × 10^−4^ ≤ ELCR), high (1.0 × 10^−5^ ≦ ELCR < 1.0 × 10^−4^), moderate (1.0 × 10^−6^ ≦ ELCR < 1.0 × 10^−5^), and low (ELCR < 1.0 × 10^−6^).

## 4. Discussion

The mean concentrations of JEM in 1980’, 1990’, and 2000~ of this study were 1.41, 0.57, and 0.06, which were comparable with the results of previous studies [7,15,19]. The occupational exposure in Korea decreased sharply around the years 1990, 1997, and 2006, when asbestos was added to the list of designated special chemicals, a ban was imposed on the use of amosite and crocidolite, and a total ban on asbestos was imposed. Hence the grouping of 4 decades could be termed reasonable. Although Korea had produced asbestos since 1930’, no exposure data including from unpublished sources was found before 1984. We estimated a two-fold higher occupational exposure before 1979 as compared to 1980’ based on a previous study which estimated occupational exposure concentration as 11.0~92.4 f/cc in 1975 which was 1.8~5-fold higher than that of 1995 [16]. Also considering the suggestion of −6.5~−7.7% annual percentage change from retrospective occupational asbestos exposure estimation studies, a two-fold estimation of ~1979 compared to 1980‘ in this study may be a conservative estimate [20]. As there was a sharp decrease in occupational exposure related to the primary asbestos industry which dealt directly with raw asbestos or produced ACMs [15], we estimated the value to remain constant to fill in the missing data for exposure under/around ACMs.

We could not perform pooled analysis for the estimation of environmental asbestos dispersion because only a few studies were conducted on environmental ambient asbestos concentration by distance from occupational exposure sources and that too without simultaneous assessment of both the inside and outside concentrations [21,22,23]. Previous studies were conducted over different periods with different atmospheric conditions in countries like Germany, Taiwan, and Indonesia. Due to the plausible meteorological differences between those studies and Korean conditions, it was not possible to use that data in the pooled analysis. The Ministry of Environment of Korea has conducted health risk assessments for the neighborhoods around former asbestos mines including NOA areas, factories including shipyards, and dense slate roof areas [24]. Hence exposure estimations of neighborhood exposure from NOA and slate roofs are available. However, the NOA and weathering situation of slate roofs may be different depending on land use and the local atmospheric conditions. Hence, we have used Korean data only for neighborhood exposure from NOA and slate roof.

The first limitation of this study is its generalizability. Due to the sparsity of data, the analysis was limited to the few available studies and the results cannot be generalized. This problem is specific to the neighborhood exposure estimation by distance from occupational exposure sources. The estimation of emission and dispersion from occupational sources to the neighborhood environment requires simultaneous exposure assessments, strong motivation, and support from the central and municipal governments and academics in the areas of both occupational and environmental health. The fact that until 2019 sixty-six countries had banned the production, use, and trade of asbestos makes further studies difficult. In addition to the above, the availability of data from only a small number of countries makes it difficult to apply this result to countries other than Korea, which is unique in having NOA and slate roofs. The second limitation is the validity issue. While this study was aimed at developing a methodology for assessment, it could not be validated. This is the first attempt to the best of the author’s knowledge, at this kind of comprehensive past exposure estimation. Hence, validity tests using various data including real assessment or experimental studies, and matching with patient or compensation data is necessary. The third limitation is applicability. Comprehensive past exposure reconstructions need a foundation of comprehensive databases (DB) including JEM and national exposure sources. Not many countries would attempt to build this DB. Also, the use of historical addresses and mobile exposure sources would need complicated calculation processes involving matching the geographical information system (GIS) and the above DB, which in turn will need the development of an appropriate computerized program.

This is one of the very few attempts that have been made to estimate the past asbestos exposure via almost all the possible routes. Identifying occupational, para-occupational, neighborhood, residence in an NOA area or under a slate roof, and household exposures might not only be used for epidemiologic purposes and compensation but will also help the public to focus on future risk prevention. The study results suggest the need and direction for further research to overcome the limitations stated above. This study also shows the need for a total ban on asbestos by describing the possible routes of exposure for ordinary people who did not directly deal in or with asbestos.

## 5. Conclusions

A nationwide past asbestos exposure assessment method for Korea was developed. The past occupational exposure reconstruction was conducted by rebuilding the previous general population JEM. Para-occupational and household exposure estimation were based on the pooled analysis of data from other countries as well as Korea. Because of meteorological differences, exposure from NOA and slate roof were estimated by a pooled analysis of Korean data only. The neighborhood exposure from occupational sources by distance was estimated by the exponential decay model. For improved generalizability, validity, and applicability of the proposed method, further studies on each route with real assessments and experiments are required. Further research on comparisons of this comprehensive estimation via various routes with other types of data including patient data and compensations is also required.

## Figures and Tables

**Table 1:** Asbestos concentrations of exposure groups without blanks in any of the 3 decades of recent Korean asbestos job-exposure matrix (JEM).

Exposure Group (EG)	Industry (KSIC, 2007)		Occupation (KSOC, 7th)		Concentration (f/cc)		
	Code	Name (KOR)	Code	Name (KOR)	1980~89	1990~99	2000~09
EG9	17129	Manufacture of Other Paper and Paperboard	89132	Paper Machine Operators	0.8097	0.0094	0.0047
EG22	20302	Manufacture of Synthetic Resin and Other Plastic Materials	83239	Plastic Products Production Machine Operators n.e.c.	0.8610	0.0431	0.0431
EG44	23911	Manufacture of Stone Products for Construction	84341	Mineral Ore and Stone Processing Machine Operators	0.4600	0.7400	0.1450
EG48	23994	Manufacture of Asbestos, Mineral Wools and Other Similar Products	821, 8221	Textile Production and Processing Machine Operators	7.4800	2.5500	0.1400
EG52	23994	Manufacture of Asbestos, Mineral Wools and Other Similar Products	8433	Cement and Mineral Products Production Machine Operators	1.700	0.7800	0.0180
EG89	30399	Manufacture of Other Parts and Accessories for Motor Vehicles n. e. c.	85429	Automobile Parts Assemblers n.e.c.	0.4200	0.4200	0.0330
EG91	31114	Manufacture of Sections for Ships	85432	Ship Assemblers	1.2300	0.0573	0.0349
EG128	85	Education	252	School Teachers	0.0004	0.0030	0.0038
EG135	95119 (50130)	Other Maintenance and Repair Services of General Machinery	75220	Ship Mechanics	0.2300	0.0060	0.1380
EG139	95212	Repair Services of Motor Vehicles Specializing in Parts	7510	Automobile Mechanics	0.9300	1.0500	0.0800
Mean (SD)					1.4120 (2.1890)	0.5659 (0.7986)	0.0641 (0.0573)
Ratio					1.0	0.5	0.05

## Appendix C. Reconstructed Korean Asbestos Job Exposure Matrix

**Table:**

Exposure Group (EG)	Industry (KSIC, 2007)		Occupation (KSOC, 7th)		Concentration (f/cc)			
	Code	Name	Code	Name	~1979	1980s	1990s	2000s
EG1	07290	Mining of Other Non-metal Ores n.e.c.	91002	Mining Laborers	0.47000	0.23500	0.11750	0.01175
EG2	07290	Mining of Other Non-metal Ores n.e.c.	83121	Chemical Material Grinding and Mixing Machine Operators	5.88000	2.94000	1.47000	0.14700
EG3	07290	Mining of Other Non-metal Ores n.e.c.	78412	Quarrymen	0.00600	0.00600	0.00600	0.00600
EG4	10301	Processing and Preserving of Fruit and Vegetables, Pickled Food	71052	Side Dish Makers	0.01300	0.01300	0.01300	0.01300
EG5	13102	Spinning of wool	8211	Textile Processing Machine Operators	0.74000	0.74000	0.74000	0.74000
EG6	13213	Weaving of Man-Made Fiber Fabrics	82211	Weaving Machine Operators	1.52000	1.52000	1.52000	1.52000
EG7	13993	Manufacture of Special Yarns and Tire Cord Fabrics	8211	Textile Processing Machine Operators	0.07300	0.07300	0.07300	0.07300
EG8	15219	Manufacture of Other Footwear	721	Textile and Leather Related Workers	0.02580	0.02580	0.02580	0.02580
EG9	17129	Manufacture of Other Paper and Paperboard	89132	Paper Machine Operators	0.80970	0.80970	0.00940	0.00470
EG10	17129	Manufacture of Other Paper and Paperboard	8914	Paper products production machine perators	1.61000	1.61000	1.61000	1.61000
EG11	17221	Manufacture of Paper Sacks and Paper Bags	84219	Painting Machine Operators n.e.c.	0.11250	0.11250	0.11250	0.11250
EG12	17222	Manufacture of Paperboard Boxes and Containers	89141	Box and Envelope Making Machine Operators	0.45180	0.45180	0.45180	0.45180
EG13	17222	Manufacture of Paperboard Boxes and Containers	84219	Painting Machine Operators n.e.c.	1.51000	1.51000	1.51000	1.51000
EG14	17902	Manufacture of Sanitary Paper Products	89144	Sanitary Paper Products Machine Operators	0.11560	0.11560	0.11560	0.11560
EG15	17909	Manufacture of Other Articles of Paper and Paperboard n.e.c.	89190	Wood and Paper Related Machine Operators n.e.c.	3.54400	3.54400	3.54400	3.54400
EG16	20111	Manufacture of Basic Organic Petrochemicals	83219	Chemical Products Production Machine Operators n.e.c.	0.01030	0.01030	0.01030	0.01030
EG17	424	Interior and Building Completion	7824	Construction Carpenters	0.46400	0.23200	0.11600	0.01160
EG18	2030	Manufacture of Synthetic Rubber and of Plastics in Primary Forms	8312	Chemical Material Processing Machine Operators	0.11280	0.11280	0.11280	0.11280
EG19	20302	Manufacture of Synthetic Resin and Other Plastic Materials	83121	Chemical Material Grinding and Mixing Machine Operators	1.06000	1.06000	1.06000	1.06000
EG20	20302	Manufacture of Synthetic Resin and Other Plastic Materials	83124	Chemical Material Distiller and Reactor Operators	0.73000	0.73000	0.73000	0.73000
EG21	20302	Manufacture of Synthetic Resin and Other Plastic Materials	84219	Painting Machine Operators n.e.c.	0.68940	0.68940	0.68940	0.68940
EG22	20302	Manufacture of Synthetic Resin and Other Plastic Materials	83239	Plastic Products Production Machine Operators n.e.c.	1.72200	0.86100	0.04310	0.04310
EG23	20421	Manufacture of General Paints and Similar Products	83121	Chemical Material Grinding and Mixing Machine Operators	0.61880	0.61880	0.61880	0.61880
EG24	20431	Manufacture of Surface-Active Agents	83213	Detergents Production Machine Operators	2.45000	2.45000	2.45000	2.45000
EG25	20493	Manufacture of Adhesives and Gelatin	83121	Chemical Material Grinding and Mixing Machine Operators	0.05450	0.05450	0.05450	0.05450
EG26	20499 (20111)	Manufacture of All Other Chemical Products n.e.c.	83219	Painting Machine Operators n.e.c.	0.01030	0.01030	0.01030	0.01030
EG27	21300	Manufacture of Pharmaceutical Goods Other Than Medicaments	83211	Pharmaceutical Products Production Machine Operators	0.01600	0.01600	0.01600	0.01600
EG28	221	Manufacture of Rubber Products	83239	Plastic Products Production Machine Operators n.e.c.	0.10970	0.10970	0.10970	0.10970
EG29	22111	Manufacture of Tires and Tubes	83221	Tire Production Machine Operators	0.65800	0.65800	0.65800	0.65800
EG30	22191	Manufacture of Industrial Un-vulcanized Rubber Products	83229	Tire and Rubber Products Production Machine Operators n.e.c.	0.96050	0.96050	0.96050	0.96050
EG31	22199	Manufacture of Other Rubber Products n.e.c.	83222	Rubber Products Production Machine Operators	0.01170	0.01170	0.01170	0.01170
EG32	20301	Manufacture of Synthetic Rubber	83222	Rubber Products Production Machine Operators	0.46840	0.46840	0.46840	0.46840
EG33	22232	Manufacture of Packaging Plastics and Shipping Containers	83231	Plastic Catapulting Machine Operators	0.00750	0.00750	0.00750	0.00750
EG34	22250	Manufacture of Foamed Plastic Products	83239	Plastic Products Production Machine Operators			5.12000	
EG35	22299	Manufacture of Other Plastic Products n.e.c.	83239	Plastic Products Production Machine Operators n.e.c.	0.01200	0.01200	0.01200	0.01200
EG36	20302	Manufacture of synthetic resin and other plastic materials	83239	Plastic Products Production Machine Operators n.e.c.	1.72400	0.86200	0.04310	0.04310
EG37	23199	Manufacture of All Other Glass and its Products n.e.c.	84319	Glass and Glass Products Machine Operators n.e.c.	0.00650	0.00650	0.00650	0.00650
EG38	23211	Manufacture of Pottery and Ceramic Household or Ornamental Ware	84321	Pottery and Porcelain Products Production Machine Operators	0.00640	0.00640	0.00640	0.00640
EG39	23229	Manufacture of Other Refractory Ceramic Products	84319	Glass and Glass Products Machine Operators n.e.c.	0.06400	0.06400	0.06400	0.06400
	23229	Manufacture of Other Refractory Ceramic Products	84322	Brick and tile molding machine operators	0.06420	0.06420	0.06420	0.06420
	23229	Manufacture of Other Refractory Ceramic Products	84399	Nonmetal Products Related Production Machine Operators n.e.c.	0.06900	0.06900	0.06900	0.06900
EG40	23239	Manufacture of Other Structural Non-refractory Clay and Ceramic Products	8432	Clay Products Production Machine Operators	0.00370	0.00370	0.00370	0.00370
EG41	23324	Manufacture of Cellulose Fiber Cement Products	84331	Cement and Lime Production Related Machine Operators	0.01340	0.01340	0.01340	0.01340
EG42	23325	Manufacture of Concrete Roofing Tiles, Bricks and Blocks	84322	Brick and Tile Production Machine Operators	0.05900	0.05900	0.05900	0.05900
EG43	2391	Cutting, Shaping and Finishing of Stone	78230	Construction Stonemasons	1.18000	1.18000	1.18000	1.18000
EG44	23911	Manufacture of Stone Products for Construction	84341	Mineral Ore and Stone Processing Machine Operators	0.46000	0.46000	0.74000	0.14500
EG45	23919	Manufacture of Other Stone Products	78230	Construction Stonemasons	0.39950	0.39950	0.39950	0.39950
EG46	23992	Manufacture of Abrasive Articles	84392	Brightener Production Machine Operators	0.80700	0.80700	0.80700	0.56000
EG47	7121	Quarrying of Monumental and Building Stone	84341	Mineral Ore and Stone Processing Machine Operators	0.91200	0.91200	0.91200	0.91200
EG48	23994	Manufacture of Asbestos, Mineral Wools and Other Similar Products	821, 8221	Textile Production and Processing Machine Operators	14.96000	7.48000	2.55000	0.14000
EG49	23994	Manufacture of Asbestos, Mineral Wools and Other Similar Products	83121	Chemical Material Grinding and Mixing Machine Operators	0.06000	0.06000	0.06000	0.06000
EG50	23994	Manufacture of Asbestos, Mineral Wools and Other Similar Products	84159	Metal Processing Machine Operators n.e.c.	0.02500	0.02500	0.02500	0.02500
EG51	23994	Manufacture of Asbestos, Mineral Wools and Other Similar Products	84322	Brick and Tile Production Machine Operators	0.03000	0.03000	0.03000	0.03000
EG52	23994	Manufacture of Asbestos, Mineral Wools and Other Similar Products	8433	Cement and Mineral Products Production Machine Operators	3.40000	1.70000	0.78000	0.01800
EG53	23999	Manufacture of Other Unclassified Non-metallic Minerals n.e.c.	84399	Nonmetal Products Related Production Machine Operators n.e.c.	0.06900	0.06900	0.06900	0.06900
EG54	24119 (24111)	Manufacture of Other Basic Iron and Steel (Manufacture of Basic Iron)	84141	Ore and Metal Furnace Operators	0.00820	0.00820	0.00820	0.00820
EG55	24121	Manufacture of Hot Rolled, Drawn and Extruded Iron or Steel Products	84151	Rolling Mill Operators	0.04000	0.04000	0.04000	0.04000
EG56	2431	Cast of Iron and Steel	84110	Metal Casting Machine Operators	3.08000	1.54000	0.77000	0.07700
EG57	25119	Manufacture of Other Structural Metal Products	84213	Metal Product Painting Machine Operators	0.21130	0.21130	0.21130	0.21130
EG58	25911 (25999)	Manufacture of Powder Metallurgic Products	84159	Metal Processing Machine Operators n.e.c.	0.11200	0.05600	0.02800	0.00280
EG59	25912 (24)	Forging of Metal/Manufacture of Basic Metal Products	74130	Forge Hammer smiths and Forging Press Workers	0.00800	0.00800	0.00800	0.00800
EG60	25913	Manufacture of Metal Pressed and Stamped Products	84151	Rolling Mill Operators	0.00670	0.00670	0.00670	0.00670
EG61	25921	Heat Treatment of Metals	84155	Metal Heat Treatment Furnace Operators	0.03370	0.03370	0.03370	0.03370
EG62	25923	Coating and Similar Treatment of Metals	84229	Plating and Metal Spraying Machine Operators n.e.c.	0.11710	0.11710	0.11710	0.11710
EG63	25934	Manufacture of Saws, Saw Blades and Interchangeable Tools	74110	Die and Mold Makers	0.00860	0.00860	0.00860	0.00860
EG64	26299	Manufacture of Other Electronic Valves, Tubes and Electronic Components n.e.c.	86321	Electronic Parts Production Equipment Operators	0.01060	0.01060	0.01060	0.01060
EG65	2642	Manufacture of Broadcasting and Wireless Telecommunication Apparatuses	86409	Electrical, Electronic Parts and Products Assembler n.e.c.	0.02800	0.02800	0.02800	0.02800
EG66	26529	Manufacture of Other Sound Equipment	86402	Audio-Visual Equipment Assemblers	0.02200	0.02200	0.02200	0.02200
EG67	27216	Manufacture of Industrial Process Control Equipment	76224	Electrical Control Unit Fitters and Mechanics	0.00100	0.00100	0.00100	0.00100
EG68	27216	Manufacture of Industrial Process Control Equipment	85101	Lathe Machine Operators	0.00200	0.00200	0.00200	0.00200
EG69	28111	Manufacture of Electric Motors and Generators	86401	Electrical Equipment Assemblers	0.01430	0.01430	0.01430	0.07180
EG70	28119	Manufacture of Other Electric Motors, Generators and Transformers	85109	Metal Work Machinery Operators n.e.c.	0.06460	0.06460	0.06460	0.07540
EG71	28119	Manufacture of Other Electric Motors, Generators and Transformers	8610, 86311	Power Generation and Distribution Equipment Operators, Electrical Parts Production Equipment Operators	0.00400	0.00400	0.00400	0.00400
EG72	28302	Manufacture of Other Insulated Wire and Cable	86402	Audio-Visual Equipment Assemblers	0.35790	0.35790	0.35790	0.35790
EG73	28303	Manufacture of Insulated Codes Sets and Other Conductors for Electricity	86401	Electrical Equipment Assemblers	0.12450	0.12450	0.12450	0.12450
EG74	28410	Manufacture of Electric Lamps and Electric Bulbs	86312	Electrical Products Production Equipment Operators	0.20310	0.20310	0.20310	0.20310
EG75	28422	Manufacture of General Electric Lighting Fixture	86401	Electrical Equipment Assemblers	0.01970	0.01970	0.01970	0.01970
EG76	28519	Manufacture of Other Domestic Electric Appliances	86312	Electrical Products Production Equipment Operators	0.00500	0.00500	0.00500	0.00500
EG77	29132	Manufacture of Pumps and Compressors	89904	Air Compressor Operators	0.00500	0.00500	0.00500	0.00500
EG78	29133	Manufacture of Taps, Valves and Similar Products	8510	Metal Work Machinery Operators	0.55600	0.55600	0.55600	0.55600
EG79	29169	Manufacture of Other Work trucks, Lifting and Handling Equipment	8544	General Machinery Assemblers	0.00900	0.00900	0.00900	0.00900
EG80	29210	Manufacture of Agricultural and Forestry Machinery	83239	Plastic Products Production Machine Operators n.e.c.	0.00260	0.00260	0.00260	0.00260
EG81	29210	Manufacture of Agricultural and Forestry Machinery	85442	Agricultural Machinery Assemblers	0.04630	0.04630	0.04630	0.04630
EG82	29250	Manufacture of Machinery for Food, Beverage and Tobacco Processing	811	Food Processing Related Machine Operators	0.00780	0.00780	0.00780	0.00780
EG83	29299	Manufacture of Other Special Purpose Machinery, n.e.c.	85441	Industry Machinery Assemblers	0.11330	0.11330	0.11330	0.11330
EG84	30121	Manufacture of Passenger Motor Vehicles	85410	Automobile Assemblers	0.02330	0.02330	0.02330	0.02330
EG85	303	Manufacture of Parts and Accessories for Motor Vehicles and Engines	74130	Forge Hammersmiths and Forging Press Workers	0.00110	0.00110	0.00110	0.00110
EG86	30310	Manufacture of Parts and Accessories for Motor Engines	85421	Automobile Engine Assemblers	0.28000	0.14000	0.07000	0.00230
EG87	30399	Manufacture of Other Parts and Accessories for Motor Vehicles n.e.c.	75105	Automobile Paint Mechanics	1.05000	1.05000	1.05000	1.05000
EG88	303	Manufacture of Other Parts and Accessories for Motor Vehicles n.e.c.	85429	Automobile Parts Assemblers n.e.c.	0.18000	0.18000	0.18000	0.18000
EG89	30399	Manufacture of Other Parts and Accessories for Motor Vehicles n.e.c.	85429	Automobile Parts Assemblers n.e.c.	0.84000	0.42000	0.42000	0.03300
EG90	31111	Building of steel ships	75220	Ship Mechanics	0.52000	0.26000	0.13000	0.13000
EG91	31114	Manufacture of Sections for Ships	85432	Ship Assemblers	2.46000	1.23000	0.05730	0.03490
EG92	31322	Manufacture of Aircraft Parts and Accessories	85433	Aircraft Assemblers	0.09500	0.09500	0.09500	0.09500
EG93	3320	Manufacture of Musical Instruments	73031	Musical Instrument Makers and Repairers	0.01850	0.01850	0.01850	0.02200
EG94	33999	Other Manufacturing n.e.c.	83124	Chemical Material Distiller and Reactor Operators	0.83550	0.83550	0.83550	0.83550
EG95	3511	Electric Power Generation	8610	Power Generation and Distribution Equipment Operators	0.00360	0.00360	0.00360	0.00360
EG96	3511	Electric Power Generation	23519	Machine Engineers and Researchers n.e.c.	0.00360	0.00360	0.00360	0.00360
EG97	36010	Collection, Purification and Distribution of Water to Household	8810	Water Treatment Plant Operators	0.06600	0.06600	0.06600	0.06600
EG98	38120	Hazardous Waste Collection	8820	Recycling Machine and Incinerator Operators	0.00310	0.00310	0.00310	0.00310
EG99	38120	Hazardous Waste Collection	91001	Construction Laborers	0.00500	0.00500	0.00500	0.00500
EG100	382	Waste Treatment Services	8820	Recycling Machine and Incinerator Operators	0.01600	0.01600	0.01600	0.01600
EG101	38220	Disposal of Hazardous Waste	88209	Recycling Machine and Incinerator Operator n.e.c.	0.01300	0.01300	0.01300	0.01300
EG102	41224	Installation of Environmental Hygience Treatment Appliances	88209	Recycling Machine and Incinerator Operator n.e.c.	0.00180	0.00180	0.00180	0.00180
EG103	41112	Apartment Building Construction	772	Broadcasting and Telecommunications Equipment Related Fitters and Repairers	0.03930	0.03930	0.03930	0.03930
EG104	41229	Other Civil Engineering Construction	23123	Building Construction Engineers	0.00420	0.00420	0.00420	0.00420
EG105	42110	Wrecking and Demolition of Buildings and Other Structures	78293	Building Demolition Workers	0.16960	0.08480	0.04240	0.00430
EG106	42121	Excavating and earthmoving	78499	Mining and Civil Engineering Related Workers n.e.c.	0.00060	0.00060	0.00060	0.00060
EG107	42132	Steel Reinforcing and Reinforced Concrete Works	7822	Concrete Placers and Assemblers	0.00100	0.00100	0.00100	0.00100
EG108	42134	Pavement Works	7836	Construction Painters	0.00100	0.00100	0.00100	0.00100
EG109	42137	Scaffolding and Frame Works	78291	Scaffolders	0.02100	0.02100	0.02100	0.02100
EG110	4521	Sale of Motor Vehicle New Parts and Accessories	52129	Store Salespersons n.e.c.	2.84000	1.42000	0.71000	0.00000
EG111	471	Retail Sale in Non-Specialized Stores	5211	Owners and Supervisors of Small Stores	0.00020	0.00020	0.00020	0.00300
EG112	47119	Retail Sale in Other Non-Specialized Large Stores	5211	Owners and Supervisors of Small Stores	0.00530	0.00530	0.00530	0.00530
EG113	501	Sea and Coastal Water Transport	8760	Ship Workers and Related Workers				
EG114	50122	Coastal freight water transport	92101	Freight Loading and Lifting Laborers				
EG115	52911	Supporting, Railway Transport Activities	31262	Railway Transport Clerks	0.00800	0.00800	0.00800	0.00290
EG116	79211/ 52911	Supporting, Railway Transport Activities	7523	Locomotive and Electric Train Mechanics	0.07200	0.03600	0.01800	0.00180
EG117	52911	Supporting, Railway Transport Activities	75232	Railroad train mechanics	1.48400	0.74200	0.37100	0.03710
EG118	52915	Operation of Vehicle Parking Facilities	52132	Passenger Ticket Salespersons	0.00390	0.00390	0.00390	0.00390
EG119	59141	Motion Picture Exhibition	28399	Drama, Film and Video Related Workers n.e.c.	0.00600	0.00600	0.00600	0.00600
EG120	6022	Broadcasting via Cable, Satellite and Other Broadcasting	2250	Telecommunication and Broadcast Transmissions Equipment Technicians	0.00500	0.00500	0.00500	0.00500
EG121	68211	Residential Property Management	85201	Cooler and Heater Related Machine Operators	0.00200	0.00200	0.00200	0.00200
EG122	95119	Other Maintenance and Repair Services of General Machinery	75351	Building Boiler Fitters and Mechanics	0.22107	0.11054	0.05527	0.00553
EG123	70129	Research and Experimental Development on Other Engineering	13114	Engineering Research Managers	0.11910	0.11910	0.11910	0.11910
EG124	72122	Environmental Consulting and Related Engineering Services	15301	Environmental Service Related Managers	0.00100	0.00100	0.00100	0.00100
EG125	74100	Business Facilities Support Management Services	12090	Public and Business Administration Managers	0.00150	0.00150	0.00150	0.00150
EG126	75290	Other Tourist Assistance and Reservation Services	52132	Passenger Ticket Salespersons	0.01000	0.01000	0.01000	0.01000
EG127	84213	Regulation of Activities of Environment Affairs	21125	Astronomy and Space Science Researchers	0.47050	0.47050	0.47050	0.47050
EG128	85	Education	252	School Teachers	0.00380	0.00036	0.00300	0.00380
EG129	85501	General Subject Educational Institute	25419	Liberal Arts and Language Instructors n.e.c.	0.00700	0.00700	0.00700	0.00700
EG130	8610 86101 86103	Hospital Activities General Hospitals	24302 24	General Nurses Health, Social Welfare and Religion Related Occupations	0.00490	0.00490	0.00490	0.00490
EG131	87210 85110	Child Day Care Services	24720	Child Care Teachers	0.00720	0.00720	0.00720	0.00720
EG132	90211	Library and Archives Activities	28221	Librarians	0.00240	0.00240	0.00240	0.00240
EG133	90221	Museum Operation	28211	Curators	0.00150	0.00150	0.00150	0.00150
EG134	91131	Other Complex Sports Facility Operation	28691	Sports Instructors and Trainers	0.00600	0.00600	0.00600	0.00600
EG135	95119 (50130)	Other Maintenance and Repair Services of General Machinery	75220	Ship Mechanics	0.46000	0.23000	0.00600	0.13800
EG136	95119 (50130)	Other Maintenance and Repair Services of General Machinery	79222	Ship Plumbers	0.48800	0.48800	0.48800	0.48800
EG137	95119	Other Maintenance and Repair Services of General Machinery	75220	Ship Mechanics	0.06240	0.06240	0.06240	0.06240
EG138	95211	General Repair Services of Motor Vehicles	75105	Automobile Paint Mechanics	0.88000	0.88000	0.88000	0.88000
EG139	95212	Repair Services of Motor Vehicles Specializing in Parts	7510	Automobile Mechanics	1.86000	0.93000	1.05000	0.08000
EG140	96121	Saunas	42234	Bathing Attendants	0.00650	0.00650	0.00650	0.00650
EG141	96991	Wedding Chapel Services	42320	Wedding Ceremony Workers	0.00400	0.00400	0.00400	0.00400
